# Man with Pleuritic Chest Pain

**DOI:** 10.5811/cpcem.2022.10.57915

**Published:** 2023-02-09

**Authors:** Tanner George Greiving, Sumeru G. Mehta

**Affiliations:** * Brooke Army Medical Center, Department of Emergency Medicine, San Antonio, Texas; † Methodist Hospital, Emergency Department, San Antonio, Texas

**Keywords:** Epipericardial fat necrosis, pericardial fat necrosis, epicardial fat necrosis

## Abstract

**Case Presentation:**

We describe a case of epipericardial fat necrosis.

**Discussion:**

Epipericardial fat necrosis is an inflammatory condition in which the pericardial fat pad necrotizes resulting in surrounding inflammation. This condition mimics more ominous pathology in clinical presentation and radiographic findings. Management is supportive with oral analgesics.

## CASE PRESENTATION

A 39-year-old male presented to the emergency department (ED) for three days of right-sided, pleuritic chest pain. The patient denied any preceding trauma or illness. Examination revealed no overlying skin changes or reproducible chest wall tenderness, although lung sounds were noted to be diminished near the right lung base. His vital signs were as follows: temperature of 98.2° Fahrenheit; respiratory rate of 17 breaths per minute; pulse oximetry of 95% on room air; blood pressure of 135/82 millimeters of mercury; and heart rate of 92 beats per minute.

**Image 1:**
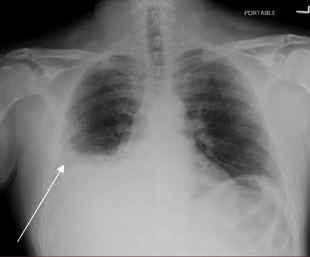
Chest radiograph. Arrow pointing at right-sided pleural effusion.

**Image 2:**
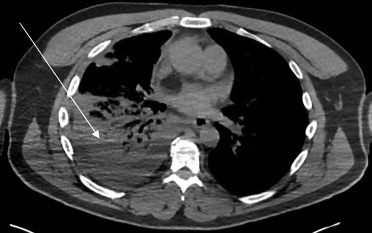
Non-contrast enhanced computed tomography of the chest. Arrow pointing at multilobular atelectasis and pleural effusion.

**Image 3:**
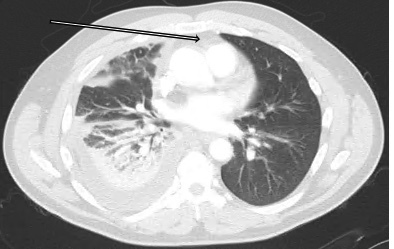
Computed tomography angiogram of the chest. Arrow pointing at an encapsulated mediastinal fatty lesion with soft tissue stranding. Other notable findings include sympathetic right pleural effusion with right lung atelectasis.

Chest radiograph revealed a right pleural effusion with right base consolidation suspicious for pneumonia (Image 1). Based on historical factors not consistent with pneumonia and discussion with the radiologist, a computed tomography (CT) chest without contrast was initially ordered. The CT chest demonstrated multilobular consolidations within the right lung with an associated moderate volume pleural effusion (Image 2). Subsequent concerns about possible pulmonary infarction as a cause of the pleural effusion prompted a CT angiogram. Computed tomography angiography demonstrated acute epipericardial fat necrosis with sympathetic right pleural effusion and right lung atelectasis (Image 3). The patient’s pain was controlled with oral analgesics during evaluation in the ED; he was then discharged home with continued oral analgesic therapy.

## DISCUSSION

Epipericardial fat necrosis is a rare benign condition^1^ that presents as acute pleuritic chest pain. The description of symptoms may reflect that of more ominous pathologies including acute myocardial infarction, pulmonary embolism, or acute pericarditis.^2^ Epipericardial fat necrosis is characterized as a self-limited inflammatory process occurring inside the epipericardial fat—the tissue connecting the pericardial layer to the anterior thoracic wall.^3^ Findings on chest radiograph are typically non-specific.^4^ Computed tomography is the imaging modality of choice for diagnosis, although a CT angiogram may be warranted to rule out pulmonary embolism.^5^ Current management is supportive centering around oral analgesia, typically non-steroidal anti-inflammatories.^3^ A follow-up, non-contrast enhanced CT should be considered at 4–8 weeks to confirm expected healing.^5^

*CPC-EM Capsule*

What do we already know about this clinical entity?

*Epipercardial fat necrosis is a self-limited, inflammatory condition which often causes chest pain and radiographic findings suggestive of more ominous pathologies.*

What is the major impact of the image(s)?

*These images demonstrate the characteristic fat pad changes in combination with radiographic findings that may also be present with more ominous pathologies.*

How might this improve emergency medicine practice?

*Early recognition of this etiology may reduce excessive imaging and aid in the initiation of appropriate management.*

## References

[1] Hernandez D, Galimany J, Pernas JC (2011). Case 170: Pericardial fat necrosis. Radiology.

[2] Pineda V, Cáceres J, Andreu J (2005). Epipericardial fat necrosis: radiologic diagnosis and follow-up. Am J Roentgenol.

[3] Giassi KdS, Costa AN, Bachion GH (2014). Epipericardial fat necrosis: an underdiagnosed condition. Br J Radiol.

[4] Jackson RC, Clagett OT, Mcdonald JR (1957). Pericardial fat necrosis: report of three cases. J Thorac Surg.

[5] Ataya D, Chowdhry AA, Mohammed TH (2011). Epipericardial fat pad necrosis. J Thorac Imaging.

